# *Staphylococcus epidermidis* meningitis in the absence of a neurosurgical device secondary to catheter-related bloodstream infection: a case report and review of the literature

**DOI:** 10.1186/s13256-018-1646-7

**Published:** 2018-04-25

**Authors:** Taro Noguchi, Miki Nagao, Masaki Yamamoto, Yasufumi Matsumura, Toshiyuki Kitano, Akifumi Takaori-Kondo, Satoshi Ichiyama

**Affiliations:** 1 Department of Clinical Laboratory Medicine, Kyoto University Graduate School of Medicine, 54 Shogoin-Kawara-cho, Sakyo-ku, Kyoto, 606-8507 Japan; 2 Department of Hematology and Oncology, Kyoto University Graduate School of Medicine, 54 Shogoin-Kawara-cho, Sakyo-ku, Kyoto, 606-8507 Japan

**Keywords:** Coagulase-negative *Staphylococcus*, Meningitis, Neurosurgical device, Catheter-related bloodstream infection, Acute leukemia, Neutropenia

## Abstract

**Background:**

*Staphylococcus epidermidis* can cause nosocomial meningitis in the presence of prosthetic devices. We describe a case of *Staphylococcus epidermidis* meningitis in a patient with neutropenia who had no intracranial foreign body, and we review the literature on meningitis that is caused by coagulase-negative *Staphylococcus* spp. without a neurosurgical device.

**Case presentation:**

A 47-year-old Japanese man with acute myeloid leukemia receiving chemotherapy through a totally implantable central venous catheter developed fever and headache. The patient had a history of craniotomy for anaplastic oligodendroglioma without an indwelling neurosurgical device. The results of two blood cultures and a cerebrospinal fluid culture were positive for *Staphylococcus epidermidis*. Clinical improvement was observed with treatment with vancomycin and removal of the central venous catheter despite prolonged neutropenia.

**Conclusions:**

To the best of our knowledge, this is the first reported case of *Staphylococcus epidermidis* meningitis in a patient with neutropenia without a neurosurgical device who was successfully treated.

## Background

*Staphylococcus epidermidis* has emerged as a major pathogen in hospital-acquired infections and is currently the most common agent of infections of intravenous catheters and indwelling prosthetic devices [1]. *S. epidermis* colonization on the patient’s skin and on the hands of health care workers is considered to be the most common source of these infections. The ability of *S. epidermidis* to adhere and form biofilms on the surface of foreign bodies is thought to be the most significant mechanism that is associated with this bacterium [2].

*S. epidermidis* meningitis is usually associated with neurosurgical devices such as ventriculoperitoneal shunts, and *S. epidermidis* meningitis in the absence of these devices is very rare. We report a case of a patient who developed *S. epidermidis* meningitis unrelated to the neurosurgical device and who was successfully treated in spite of profound and prolonged neutropenia.

## Case presentation

Our patient was a 47-year-old Japanese man who had been diagnosed with acute myeloid leukemia in January 2013. He received induction chemotherapy, experienced complete remission, and subsequently received postremission chemotherapy in March of that year. He had a totally implantable central venous access port (TICVAP) that was used for intravenous access for chemotherapy. In April, a second course of postremission chemotherapy was begun, which resulted in prolonged pancytopenia. He then received antimycotic prophylaxis with micafungin. On day 14 after initiation of chemotherapy, he developed fever, headache, vomiting without nuchal rigidity, and alteration of his mental status.

His medical history included a diagnosis of anaplastic oligodendroglioma that had been treated using a craniotomy for tumor resection in 2006 and multiple cycles of chemotherapy. No neurosurgical device was left in place after the surgery. After the surgery, the patient had left-sided hemiparesis and occasional seizures that had not been observed during the last few months. His medications were anticonvulsants (zonisamide, phenytoin, and lamotrigine) and famotidine. The patient lived with his parents and his brother in an urban area. He did not work. He consumed alcohol in moderation and did not smoke or use illicit drugs. At the time of presentation, he was alert. His temperature was 38.4 °C, his pulse was 76 beats per minute, his blood pressure was 124/64 mmHg, and his oxygen saturation was 97% while he was breathing ambient air. A cardiopulmonary examination showed no murmurs and revealed clear lung fields to auscultation bilaterally. An abdominal examination revealed a soft, nontender abdomen with no organomegaly. His muscle strength was 4 in his left limbs and 5 in his right limbs. No seizure or nuchal rigidity was observed.

**Fig. 1:**
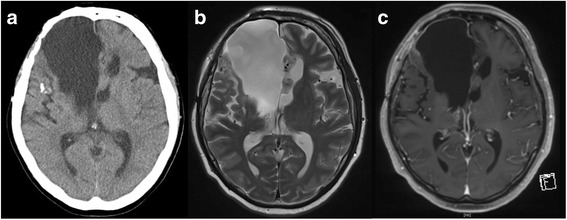
**a** Brain computed tomography showing a surgical cavity following resection of the tumor in the right frontal lobe and calcification. Magnetic resonance imaging showing the same cavity on a T2-weighted axial image (**b**) and no ring-enhancing lesions suggesting a brain abscess on a gadolinium-enhanced, T1-weighted axial image (**c**)

The patient’s laboratory data at presentation were a white blood cell (WBC) count of 0.2 × 10^9^/L, an absolute neutrophil count of < 0.1 × 10^9^/L, platelet count of 34 × 10^9^/L, and an elevated C-reactive protein level (8.3 mg/dl). The results of his renal function test were normal, as were his blood levels of electrolytes and serum aminotransferases of aspartate aminotransferase, alanine aminotransferase, and alkaline phosphatase. Computed tomography of the brain showed a surgical cavity following resection of the tumor (Fig. 1a). Two sets of blood cultures were obtained: one from the TICVAP and another from a peripheral vein according to the guidelines of the Infectious Diseases Society of America [3]. The patient was treated empirically with intravenous meropenem (1 g every 8 hours) and vancomycin (1 g every 12 hours), and the following day, a lumbar puncture was performed after platelet transfusions. The lumbar puncture yielded clear cerebrospinal fluid (CSF) with < 11 cells /μl (differential not determined). The day when the lumber puncture was done was a holiday, and CSF cell counting was performed only by an automated cell counter (XT-4000i; Sysmex, Kobe, Japan), the cutoff value of which was 11 cells/μl in our hospital. The patient’s CSF glucose was normal (3.3 mmol/L), and his protein was slightly elevated (1.7 g/L). Gram staining and India ink staining of the CSF did not show any microorganisms, and no blast cells were found in the patient’s CSF. Gadolinium-enhanced magnetic resonance imaging (MRI) of the brain showed a surgical cavity following resection of the tumor in the right frontal lobe (Fig. 1b and c).

Gram-positive cocci grew in the two blood cultures obtained on day 14 and in the one CSF culture obtained on day 15, respectively, and the patient’s TICVAP, which was considered to be the source of his bacteremia, was removed. Although the patient’s headache and vomiting partially resolved, his fever persisted and the results of repeat blood cultures were negative. Methicillin-resistant *S. epidermidis* was isolated from the patient’s blood, CSF, and catheter cultures, and the isolates were susceptible to vancomycin; therefore, intravenous vancomycin treatment was continued. Oral rifampin was added, and granulocyte colony-stimulating factor was initiated. On day 22, the patient’s headache and vomiting worsened; therefore, a lumbar puncture was performed again. The patient’s CSF total cell count yielded 130 cells/μl (83% mononuclear cells, 17% polymorphonuclear cells), his glucose level was 2.1 mmol/L, and his protein level was 4.0 g/L. On the same day, a complete blood count revealed an increased absolute neutrophil count of 1.6 × 10^9^/L. Follow-up brain MRI using gadolinium showed no significant change, and no organisms grew in the CSF culture. A third lumbar puncture was performed on day 28 and revealed improved CSF parameters (cell count 21 cells/μl, glucose 2.9 mmol/L, and protein 2.3 g/L), and the result of a CSF culture was negative. The simultaneous absolute neutrophil count was 1.3 × 10^9^/L. The patient’s symptoms continued for 3 weeks following the initiation of therapy, and antibiotic treatment was ended on day 39. No signs of relapse of meningitis were observed, and the patient was transferred to a rehabilitation facility on day 56. Three months later, his leukemia relapsed, and he died shortly after that.

The vancomycin levels in the blood and CSF were measured during treatment. Four vancomycin serum trough levels were within the therapeutic range of 11–18 mg/L, and the peak level in the serum on day 26 was 28.6 mg/L. The CSF concentration of vancomycin 1 hour after intravenous administration was 12.8 mg/L, and the CSF trough level was 9.4 mg/L.

## Discussion

We describe a case of a patient with *S. epidermidis* meningitis secondary to catheter-related bloodstream infection (CRBSI) that was unrelated to a neurosurgical device. *S. epidermidis* meningitis usually occurs as an infection related to neurosurgical devices [1]. Although our patient had neutropenia induced by chemotherapy against acute leukemia that could have led to a deteriorating prognosis of *S. epidermidis* meningitis [4], he was successfully treated with vancomycin and removal of an intravascular catheter.

Meningitis due to *S. epidermidis* infection in adults has been associated nearly exclusively with neurosurgical procedures and devices, but also with head trauma. In a previous review of 14 cases of meningitis caused by coagulase-negative *Staphylococcus* (CoNS), all cases had a postneurosurgical state upon insertion of neurosurgical devices, and the median interval between the last neurosurgical procedure and CoNS meningitis was 10.5 days (range 3–180 days) [5]. Although our patient had a history of neurosurgical procedures, 7 years had passed since his last neurosurgery, and no intracranial device was implanted. In our patient’s case, it is unlikely that meningitis developed from a primary surgical site infection. Although two patients with CoNS meningitis without neurosurgical procedure were reported in another case series, detailed information about those patients was not provided [6]. Table 1 lists the limited reports of cases of CoNS meningitis that were unrelated to neurosurgical devices [4, 7–9]. All of these patients had an underlying condition that compromised immunity, and an indwelling foreign body other than an intracranial device was left in place in all patients, but information regarding a nonneurosurgical devices was not provided for the pediatric patients. Our patient fulfilled the diagnostic criteria for CRBSI [10], and we believe that he developed a CRBSI due to *S. epidermidis* and subsequently developed meningitis.

**Table 1:** Characteristics of cases of coagulase-negative *Staphylococcus* meningitis without neurosurgical devices

Patient no.	Age^a^, sex	Underlying conditions	CoNS species	Origin of infection	Repeated positive CSF culture	Bacteremia status	Treatment/outcome	Reference
1	20, F	CML, BMT, neutropenia	*S. epidermidis*	CRBSI	Yes	Yes	Vancomycin/death on day 7	Guiot *et al*. (1994) [4]
2	18 days, F	ELBW, intraventricular hemorrhage	*S. epidermidis*	NA	Yes	No	Vancomycin/recovery	Drinkovic *et al*. (2002) [9]
3	20 days, F	ELBW, intraventricular hemorrhage	*S. capitis*, *S. warneri*	NA	Yes	No	Vancomycin, rifampicin/recovery	Drinkovic *et al*. (2002) [9]
4	65, F	Diabetes, cirrhosis, indwelling urinary catheter	*S. capitis*	Urinary tract infection?	No	One positive blood culture	Vancomycin/death on day 10	Oud (2011) [7]
5	11 months, M	Transient decreased T-helper lymphocyte absolute count, transient hypogammaglobulinemia	*S. intermedius*	Unidentified	No	NA	Cefotaxime/recovery	Durdik *et al*. (2010) [8]
6	47, M	AML, oligodendroglioma, neutropenia	*S. epidermidis*	CRBSI	No	Yes	Vancomycin, rifampicin/recovery	Our patient

*Abbreviations*: *CoNS* Coagulase-negative *Staphylococcus*, *CSF* Cerebrospinal fluid, *F* Female, *M* Male, *CML* Chronic myeloid leukemia, *BMT* Bone marrow transplant, *CRBSI* Catheter-related bloodstream infection, *ELBW* Extremely low birth weight, *AML* Acute myeloid leukemia, *NA* Not available

^a^Ages are in years unless otherwise noted

In the absence of an indwelling neurosurgical device, CoNS isolated from CSF obtained by lumbar puncture is generally considered a contaminant. Durand *et al*. proposed CoNS as an etiologic agent only when found repeatedly, and this criterion was used by other researchers [5, 11]. However, lumbar puncture is usually deferred owing to thrombocytopenia in patients with hematological malignancy. In our patient, the first CSF grew CoNS, although a Gram stain of the CSF showed no organisms. This might have been due to the administration of vancomycin prior to lumbar puncture and low inoculum of CoNS [12].

In bacterial meningitis of patients with neutropenia, *Streptococcus pneumoniae*, *Staphylococcus aureus*, *Pseudomonas aeruginosa*, and *Escherichia coli* are considered to be common etiologic pathogens [13–15]. To date, only one other documented case of neutropenic meningitis caused by *S. epidermidis* has been reported [4], and it was unfortunately fatal.

In a published series of 77 patients with meningitis who also had cancer, fever was the most frequent symptom, and headache was the second most common. In patients with neutropenia, the most frequently mentioned symptom was headache [14]. A review of 43 cases of neutropenic meningitis revealed that the most consistent clinical signs were fever and alterations in mental status, but headache was uncommon [15]. Nuchal rigidity was rare in these reviews. As such, compared with patients with meningitis in the nonneutropenic population, those with neutropenia did not demonstrate as many symptoms. The CSF WBC counts of patients with neutropenia were considerably lower than those in the nonneutropenic population [14–16]. In our patient, the only abnormal CSF finding from the initial lumbar puncture was an elevated protein level. The subsequent lumbar puncture revealed an elevated CSF cell count with a recovery of peripheral blood neutrophils.

In a previous study of CoNS meningitis, the 30-day mortality was 14% after intravenous vancomycin treatment and removal of the neurosurgical device [5]. CoNS meningitis in two adult patients without a neurosurgical device was fatal (patients 1 and 4 in Table 1). One patient, who had neutropenia and was treated without central venous catheter removal, died on day 7 after initiation of antibiotic therapy. Our patient was successfully treated with intravenous vancomycin, oral rifampin, and removal of the central venous catheter; however, he ultimately died of underlying leukemia.

## Conclusions

A literature review revealed that CoNS meningitis without a neurosurgical device has rarely been reported. No symptom or sign is reliably present in patients with neutropenia, and a high index of clinical suspicion is essential for prompt diagnosis. Because CoNS meningitis could be life-threatening, especially in patients with neutropenia, adequate antibiotic therapy with source control is critical for a successful outcome.

## Abbreviations

AML: Acute myeloid leukemia
BMT: Bone marrow transplant
CML: Chronic myeloid leukemia
CoNS: Coagulase-negative *Staphylococcus*
CRBSI: Catheter-related bloodstream infection
CSF: Cerebrospinal fluid
ELBW: Extremely low birth weight
F: Female
M: Male
MRI: Magnetic resonance imaging
NA: Not available
TICVAP: Totally implantable central venous access port
WBC: White blood cell
